# Gender Diversity, Gender Dysphoria/Incongruence, and the Intersection with Autism Spectrum Disorders: An Updated Scoping Review

**DOI:** 10.1007/s10803-024-06650-6

**Published:** 2024-12-04

**Authors:** Hannah M. Rea, Roald A. Øien, Sara Jane Webb, Shivam Bansal, John F. Strang, Anders Nordahl-Hansen

**Affiliations:** 1 Psychiatry and Behavioral Science Department, University of Washington, Seattle, WA 98195 USA; 2 UiT – The Arctic University of Norway, PB 6060, 9037 Tromsø, Norway; 3 Child Study Center, Yale School of Medicine, New Haven, CT 06510 USA; 4 Gender and Autism Program, Children’s National Hospital, Washington, DC 20010 USA; 5 Department of Education, ICT and Learning, Østfold University College, B R A Veien 4, Halden, Norway

**Keywords:** Autistic, Autism, Gender dysphoria, Gender diversity, Gender incongruence

## Abstract

In several ways, 2018 represents a global pivot point in research and care related to the intersection of gender diversity and autism, reflected by the increase in the number of studies on the intersection and broadening of topics. We conducted a scoping review of the literature published between September 2018 and January 2024 to summarize recent research in the field. We compared and contrasted research trends post September 2018 to research that was summarized in previous scoping reviews (Nordahl-Hansen et al. in J Autism Develop Disord 49:1745–1748, 2019; Øien et al. J Autism Develop Disord 48(12):4028–4037, 2018. 10.1007/s10803-018-3843-z). We also summarized systematic reviews and meta-analyses published after 2018. Compared to previous reviews, we included more comprehensive search terms to focus on gender diversity broadly, rather than just gender dysphoria or gender identity disorder, and we included more detailed coding categories to uniquely identify shifts and continuities in research priorities over time. A total of 99 empirical research studies were published after August 2018 (the end of the search in the previous review) and through January 2024 on autism, gender diversity, and related topics were reviewed. There has been growth in the quantity and quality of studies on the intersection of autism and gender diversity, although methodological improvements are still needed and described in the current review. Future research should include more collaborations with stakeholders to set research priorities that are reflective of the communities being described.

**Supplementary Information:**

The online version contains supplementary material available at 10.1007/s10803-024-06650-6.

## Foreword

“Gender diverse” has emerged as an umbrella term for describing gender identities, expressions, or experiences that differ from the stereotypes and cultural norms associated with one’s designated gender^1^ at birth (The World Professional Association for Transgender Health, (WPATH) 2022). Gender dysphoria is the psychological distress related to a discordance between gender identity and assigned gender at birth (APA, 2013). Gender Incongruence is the experience of incongruence between gender identity and the assigned gender at birth. Gender Dypshoria and Gender Incongruence as clinical designations are often inappropriately conflated with gender diversity. Gender Dysphoria and Incongruence are employed in clinical practice to facilitate access to needed gender affirming supports (e.g., psychosocial and/or medical) in order to address/reduce dysphoria or incongruence (ICD-11 Beta Draft; World Health Organization, 2021). Researchers reporting on the intersection of autism and gender diversity have not consistently employed or recognized the full implications of gender-related language and affirming terms, likely because some come from training and experiential backgrounds that are distant from the field of gender development. Thus, while we acknowledge that gender diversity, transgender identity, gender dysphoria, and gender incongruence are not the same, we provide a glossary of related terms and acknowledge and include research on gender dysphoria and incongruence to ensure the full breadth of related research is acknowledged. We, however, have selected the term “gender diversity”, in accordance with the naming standards for the new revision of the Standards of Care through the WPATH (The WPATH, 2022), as inclusive of the broad range of experiences related to gender-diverse identities and expressions in order to support nomenclature for the field moving forward.

## Introduction

Gender diversity is estimated to be present for between 1.2 and 2.7% of the general population (Zhang et al., 2020). While rates of gender diversity appear to have increased, it has been argued that increasing societal awareness has allowed a greater number of individuals to reflect on and report their gender diversity (Arnoldussen et al., 2020). The increasing research attention to gender diversity has also highlighted the proportional over-representations of gender diversity in autistic populations and autism in gender diverse populations (Kallitsounaki & Williams, 2022a, 2022b; Warrier et al., 2020).^2^

Oral histories from providers serving gender diverse youth indicated that the common intersection of gender diversity and autism was discussed clinically since at least the earliest years of the twenty-first century (J. Strang, L. Edwards-Leeper, A. de Vries, personal communication, 2023a). Starting in the second decade of this century, there has been a rapid proliferation of research documenting the link between gender diversity and autism, led first by a hallmark 2010 study by de Vries and colleagues, which reported a proportional over-representation of autism among gender diverse children and adolescents. Notably, this study was conducted in The Netherlands, a country known for its socio-culturally progressive inclusion of gender diverse individuals, as well as its pioneering model of gender care (Bussemaker, 1998; Ramos, 2008).

Since this first study, diagnoses of autism have been found to occur in 1.2–68% of individuals identified as transgender or as being gender diverse (review in Kallitsounaki & Wiliams, ). In other words, rates of autism diagnoses may be 3.03–10 times more common in gender-diverse samples compared to the general population (de Vries et al., 2010; Warrier et al., 2020). A recent meta-analysis reported that, across studies, 11% of individuals with gender dysphoria/incongruence were autistic (Kallitsounaki & Williams, 2022a, 2022b), albeit with very wide prediction intervals of prevalence estimates due to differences in assessments, cut-offs on assessments, heterogeneity in presentation, and other unidentified factors that still need to be explored.

Gender diversity and incongruence have been found to occur at higher rates in autistic people (adolescents through adults) compared to the general population (Van der Miesen et al., 2018a, 2018b), with some reporting 13–15% of autistic individuals identified as transgender, gender diverse and/or as experiencing gender dysphoria (Bretherton et al., 2021; Walsh et al., 2018) These studies generally reported on samples of autistic individuals without intellectual disability (ID). No information is available on whether the proportional over-representation is present for those with ID. In addition to the proportional overlap, the intersection of neurodivergence and gender diversity appeared to drive a range of clinical needs, some unique to the intersection (Strang et al., 2018a, 2018b, 2018c, 2018d); for example, a subset of autistic transgender youth reported barriers accessing gender care due to assumptions by family members and/or clinicians that their gender may be inauthentic and the result of autism-related neurodivergence.

In light of research identifying links between gender diversity and autism, and, given the clinical characteristics of this intersectional experience, a scoping review of studies on autism and gender dysphoria was conducted in 2018. The review identified 47 studies published between 1946 and April 2018 (Øien et al., 2018); corrections published in October 2018 reported 6 additional studies that were published between April 2018 and August 2018 (Nordahl-Hansen et al., 2019).

The publication year of the previous review, 2018, represents in several ways a global pivot point in research and care related to the common intersection of gender diversity and autism. For example, in 2018, the Autism Women’s Network (AWN) first recognized the common gender diversity and autism intersection in its constituency, and officially changed its name to the Autistic Women & Nonbinary Network (Network AWN, 2023). This shift reflects broader trends in the late 2010’s, in which the autistic community and autism-related providers began publicly acknowledging the common autism/gender diversity overlap (Autistic Self Advocacy Network, 2016; Strang et al., 2018a, 2018b, 2018c, 2018d). 2018 also heralded major developments within the World Professional Association for Transgender Health (WPATH), the international body which determines the standard of care for transgender people globally. The WPATH Global Education Institute (GEI) first included specific training on the common intersection of autism and gender diversity at the 2018 conference in Buenos Aires, Argentina (WPATH, 2018). Since that time, WPATH has continued to expand its international training through GEI, with modules specifically dedicated to the intersection of gender diversity and autism embedded in two of its primary training tracks. WPATH has also launched a neurodiversity-specific workshop (WPATH, 2022).

Since 2018 there have also been sociopolitical shifts in the United States and parts of Europe accompanied by numerous political and legislative actions affecting transgender individuals’ participation in society and access to care (Jackson et al., 2019; Savage, 2020; Swedish National Board, 2022; The Guardian, 2021). Some of this legislation is specifically tied to the autism and gender diversity intersection. This legislation includes legal actions restricting autistic transgender people from accessing care (AR Act 274, 2023), as well as laws that ban care for all transgender youth, justified in part by the over-representation of autism in transgender people (GA SB140, 2023).

Not surprisingly, given these striking shifts since 2018, the number of studies and topics covered has grown rapidly, as have gender-related clinical referrals (Expósito-Campos, et al., 2023; Jones et al., 2022). Included among the more recent publications are several systematic reviews or meta-analyses on specific topics related to the intersection of autism and gender diversity that were published during or after 2018. This current review, in contrast, provides a scoping review of the published literature. Compared to the other reviews, we utilized the most comprehensive search terms. Instead of focusing on gender dysphoria or gender identity disorder, as many reviews have done, we focused on gender diversity more broadly as many individuals who identify as gender diverse do not experience dysphoria. Further, our search terms related to neurodivergence were more inclusive than previously published reviews. Unlike other published reviews, we did not restrict our search to studies published in English. This review is also distinctive in its codification of identity characteristics within each study included in the review. Specifically, we captured the proportional representation of genders, assigned gender at birth, gender diversity status (i.e., gender diverse v. cisgender), and autism status (i.e., autistic v. not autistic) across the studies. Our broad search and detailed coding uniquely identify shifts and continuinities in research priorities over time.

This current review is also an update to previous scoping reviews that were published in 2018 (Nordahl-Hansen et al., 2019; Øien et al., 2018). As part of this current review, we were able to compare and contrast research trends prior to and post mid 2018, by comparing codings between the current review (which picks up immediately following the time point of the original 2018 reviews) and the previous reviews. As noted earlier, 2018 represents in several ways a pivot point for autistic transgender people in terms of broader community awareness and acceptance, as well as the beginning of trends towards greater restrictions for these individuals. A primary focus of this review was to employ relatively current, more inclusive language and conceptualizations regarding gender diversity and autism.

## Methods

### Literature Search

The literature search for this update was completed on February, 26, 2024 by one of the last authors. There were no date restrictions set for the search, given the updated search terms from the last review. However, only articles published after August 2018 were included in the main results below (described in more detail in Supplementary Materials Table S1), to eliminate overlap in findings between the current review and the previous ones. Studies published prior to August 2018 that were not identified in the previous reviews with the previous search terms (Øien et al., 2018; Nordahl-Hansen et al., 2019) are included in supplementary materials Table S2.

The same databases as for the initial review were used: EMBASE, MEDLINE, PubMed, PsycINFO and ERIC. The search string for databases including Boolean operators used for the current review were: Transsex* OR transgender* OR gender dysphori*OR gender identity disorder* OR gender identit* OR sexual identit* OR gender diver* OR *nonbinary* OR *non-binary* OR gender vari* OR gender nonconform* OR gender non-conform* OR sex reassign* OR transma* OR transfem* OR gender var* OR gender incongruen*AND Pervasive development* disorder* OR pdd OR pdd-nos OR pervasive developmental disorder not otherwise specified OR autis* OR Autism Spectrum Disorder* OR Asperger* OR ASD OR neurodiver* OR autism spectrum* OR autis* trait* OR autism spectrum condition*. To indicate the broader scope of the current and updated review we have added italics to the search-terms that are were not present in the 2018-review. Again, we recognize many of these terms are outdated, but had inclusive language in order to capture all studies on the topic. The following search-terms, with asterisks to include variations of wording, were added that expand beyond the 2018-review: “neurodiversity”, “gender diversity”, “nonbinary,” “gender variance,” “gender nonconformity,” “sex reassignment,” “transmasculine,” “transfeminie,” and “gender incongruence.”. This was done to more completely reflect gender diversity and its terminology. References for recent reviews were also searched. The review was conducted in accordance with guidelines for scoping reviews provided by PRISMA (Tricco et al., 2018) and Peters et al. (2020).

### Inclusion and Exclusion Criteria

Included in the search was any publication that presented empirical research on any number of participants, related to autism and gender diversity, as indicated in the title and/or abstract. We included only studies that were published in scientific peer reviewed journals. Systematic reviews and meta-analyses were not included in the primary results but are presented as they are important contributions to the field. Reviews that were not systematic (e.g., commentaries/letters to the editor, book chapters, and conference abstracts) were excluded after the title and abstract screening. No language restrictions were set according to Cochrane guidelines (Higgins et al., 2019). Studies were included in the results section if they were published after August 2018 (i.e., the end date for the search in the previous review) and not included in the previous review. Studies that were published during or prior to August 2018 with the additional search terms are included in supplementary materials for this study but are not included in the results or conclusions of the main manuscript as the goal of the study was to review recent research. We coded the same categories from the original review: age, country of origin, and research team country of origin. The current review also added the following coding categories: gender identity of participants, exclusion criteria, number of participants who had transitioned or were transitioning, autism measure used, and gender measure used.

### Screening, Study Selection, and Review Protocol

**Fig. 1:**
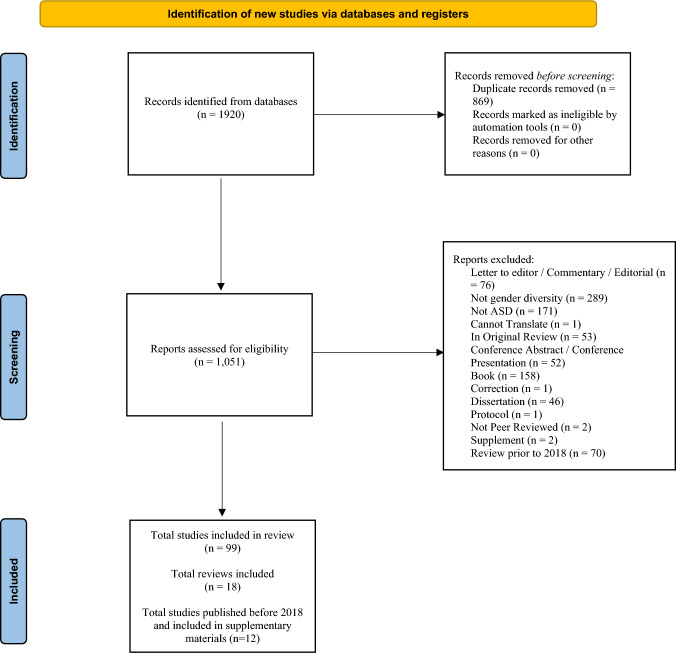
PRISMA 2020 flow diagram for updated systematic reviews which included searches of databases and registers only

Articles were screened in two stages. The first and last author screened the title and abstracts of the publications. In nine instances uncertainty was discussed and seven of these studies were included. The first author inspected and coded the full-texts using the criteria that are presented in Supplementary Materials Table S1. Questions were resolved through discussions and double-coding (done by the first and last author). See the full PRISMA flow diagram in Fig. 1. For coding categories that were consistent between the previous reviews and the current one, studies published prior to August 2018 that were ascertained in Øien et al., (2018) and Nordahl-Hansen et al. (2019) were compared to articles ascertained for the current review (i.e., published August 2018 and through January 2024) using Fisher’s exact test due to small cell sizes.

## Results

After removing duplicates, the search produced 756 results. All titles and abstracts were screened, and 6 sysematic reviews/meta-analsyes as well as 99 articles that were published after August 2018 and through January 2024 met inclusion criteria. An additional 21 articles were identified that were published during or before August 2018 with the new search criteria. Thus, the main analyses and results are based on 6 systematic reviews described in Table 1, 99 articles described in Table 2, and 12 articles presented in Supplementary Materials Table S2.

**Table 1:** Systematic reviews between 2018 and 2024

Review	Topic	Focused on the intersection of autism and gender diversity?	Search terms	Types of studies included	Number of overlapping articles with the current review	Number of articles total
Thrower (2020)	Systematic review of hypotheses, observations, and recommendations related to gender + autism	Yes	‘gender dysphoria’ OR ‘transgender’ OR ‘gender incongruency’ AND ‘autism spectrum disorder’ OR ‘autism’	Quantitative studies about prevalence	8	29
Dubreucq and Dubreucq (2021)	Review needs for care for autistic women, existing psychosocial treatments for autistic women, and strengths and limitations of the current body of research	No	“sexu^*^ “ OR “romantic relationship” OR “intimate relationship” OR “parent^*^” OR “reproductive health” OR “mother^*^” OR “pregn^*^” AND “women” OR “gender diverse” OR “transgender” OR “non-binary” AND “autism” NOT “valproate” NOT “22q11”	Quantitative, qualitative, or mixed-methods studies about autistic women’s outcomes and report on romantic relationships or parenting	4	27
Frew et al. (2021)	Identify methods used and methodology quality in research on the prevalence of psychiatric co-occurring conditions for those with “gender dysphoria”	Yes	(“gender dysphoria” OR “gender identity”) AND (comorbid* OR psychopathology) AND psych* AND child*	Quantitative and Qualitative studies on youth younger than 12 years with diagnoses of gender dysphoria	0	15
Hassrick et al. (2021)	Review literature on how autistic people use “information and communication technology”	No	[(autis* OR “pervasive developmental disorder” OR pdd OR asd) AND (internet OR “social media” OR “computer-mediated communication” OR ICT OR ict)]	Quantitative, qualitative, or mixed-methods studies on how autistic individuals use the internet to communicate	0	32
Huys and Dhondt (2021)	Review literature on the presentation of autism and gender diversity in youth	Yes	‘autism, autism spectrum disorder, neurodiversity, asperger disorder, gender dysphoria, gender non-conformity, gender diversity, gender variance, gender identity disorder, transgender, transgenderism, cross genter, adolescence’	Quantitative and qualitative studies on autistic characteristics among gender diverse youth or gender identity in autistic youth	1	12
Kallitsounaki et al. (2021)	Part 1: review of literature on co-occurrence of autism/gender diversity; Park 2: Prevalence of autism diagnosis in gender diverse populations	Yes	autism, autism spectrum disorder, autistic traits, autistic, ASD, Asperger syndrome, GD, transgender, gender dysphoric, GID, transsexualism, transgenderism, sex reassignment, GI, non-binary/nonbinary, gender variance, gender non-conformity/gender nonconformity, and gender diversity	Quantitative studies about prevalence	23	47
Lai et al. (2022)	Estimate the prevalence of co-occurring mental health diagnoses in autistic individuals	No	“autis*”, “mental health”, “psychiatr*”, “comorbid*”, “co-occurring”, and “disorder” (see supplementary materials of original article for full search terms)	Quantitative studies on prevalence of co-occurring conditions in autistic individuals	0	96
Manjra and Masic (2022)	Analyze methodologies used by quantitative studies to evaluate autism and gender diversity in children and adolescents	Yes	gender (identity OR identity disorder OR diverse OR dysphoria OR variance OR incongruence), AND autism spectrum (autism spectrum condition* OR ASC OR autism spectrum disorder* OR ASD OR Asperger syndrome OR pervasive developmental disorder not otherwise specified OR PDD-NOS OR childhood disintegrative disorder), AND age (child* OR adolescent OR young person OR teenager)	Quantitative studies on children/adolescents with a focus on reviewing methodologies	4	15
Moore et al. (2022)	Thematic metasynthesis regarding the intersection of autism and gender in qualitative research	Yes	autis* OR asperger* OR neurodivers* OR ASD OR ASC OR aspie AND Male OR female OR man OR woman OR men OR women OR boy* OR girl* OR *gender OR gender* OR feminin* OR masculin* OR transgender OR “trans sexual” OR transmasculine OR transfeminine OR “non binary” OR nonbinary OR *queer OR queer* OR intersect* OR feminist AND identit* OR meaning* OR experience* OR narrative* OR discourse* OR account* OR explor*	Qualitative studies focused on identity formation, including on how autism and gender identity impact identity formation	10	12
Mutluer et al. (2022)	Investigate rates of psychiatric comorbidities in autistic populations	No	(((("autism spectrum disorder" OR "autistic traits" OR autism OR "autistic disorder") AND (child OR pediatric OR adolescen*)) AND (epidemiolog* OR "population study")) AND (ADHD OR ''attention deficit hyperactivity disorder'' OR ''anxiety disorder'' OR depressi* OR schizo* OR ''bipolar and related disorder'' OR bipolar OR ''obsessive–compulsive and related disorder'' OR OCD OR disruptive OR impulse-control OR conduct OR sleep–wake OR ''sleep disorder'' OR ''trauma and stressor-related disorder'' OR PTSD OR ''substance-related and addictive disorder'' OR ''substance use'' OR ''gender dysphoria'' OR ''disruptive mood dysregulation syndrome'' OR DMDD OR food OR eating OR ''social phobia'' OR ''social anxiety'' OR ''oppositional defiant disorder'' OR ODD OR ''intellectual disability'' OR ''mood disorder'' OR ''feeding and eating disorder'' OR ''feeding disorder'' OR ''eating disorder'' OR ''trauma and stress related disorder'' OR ''communication disorders'' OR ''tic disorders'' OR tourette OR ''elimination disorders'' OR ''nonorganic enuresis'' OR ''non organic enuresis'' OR ''non organic encopresis'' OR ''nonorganic encopresis'' OR enuresis OR encopresis OR ''manic disorder'' OR ''Panic disorder'' OR ''panic attack'' OR ''acute stress reaction'' OR ''conversion disorder'' OR ''somatoform disorder'' OR ''somatization disorder'' OR insomni* OR hypersomni* OR sleepwalk OR ''sleep walk'' OR somnambulism OR ''sleep terror'' OR ''night terror'' OR nightmare OR ''impuls* disorder'' OR trichotilloman* OR ''gender ident* disorder'' OR ''speech disorder'' OR ''language disorder'' OR ''reading disorder'' OR ''spelling disorder'' OR ''arithmetic disorder'' OR scholastic OR ''hyperkinetic disorder'' OR mutism OR pica OR stutter* OR ''global developmental delay'' OR cataton* OR ''body dysmorphi* Disorder'' OR ''hoarding disorder'' OR ''hair pulling disorder'' OR excoriation OR ''skin picking disorder'' OR ''functional neurological symptom disorder'' OR ''rumination disorder'' OR ''food intak* disorder'' OR ''hypersomnolence disorder'' OR narcolep* OR parasomn* OR ''Restless Legs Syndrome'' OR ''intermittent* explosive disorder'' OR suicid* OR psychosis OR psychotic OR agoraphobia OR phobia OR ''anorex* nervos* '' OR bulimia OR bulimic OR ''binge eating disorder'')) AND (("2015/05/01"[Date-Publication]: "2020/05/31"[Date-Publication]))	Quantitative and qualitative research on psychiatric comorbidities in autistic children and adolescents	0	41
Pinna et al. (2022)	Review the literature on mental health conditions for transgender individuals	No	“gender dysphoria” OR “transgender persons,” AND “mental disorders.” Thereafter, the original search was expanded by substituting “mental disorders” with the following terms: “personality disorders,” “suicide,” “anxiety,” “depression,” “autism,” and “eating disorders.”	Quantitative or qualitative research on mental conditions among transgender adults	4	165
Wattel et al. (2022)	Theories on the link between autism and "trans gender modality"	Yes	ASC terms (autism, autism spectrum disorder, autis*, and asperger*) and TGM terms (gender dysphoria, gender identity disorder, transgender*, and transsex*). Search terms were combined using Boolean operators “AND” and “OR.”	Quantitative and qualitative studies focused on theories about why autism and gender diversity overlap	21	36
Bouzy et al. (2023)	Systematic review of hypotheses, observations, and recommendations related to gender and autism	Yes	’gender dysphoria’ OR ‘transgender’ OR ‘gender incongruency’ AND ‘autism spectrum disorder’ OR ‘autism’	Quantitative and qualitative studies on formally, clinically diagnosed autistic individuals with gender diverse identities	42	77
Gagnon et al. (2023)	Define challenges encountered by health professionals who work with individuals at the intersection of autism and “gender dysphoria”	Yes	1. Autis*, ASD, Asperger, Asperger syndrome, autistic people 2. Neurotypical, normal develop* neurotypical*, typical* develop* 3. Gender, gender identity, sex role, gender role, gender relations	Quantitative and qualitative studies on interventions with autism or neurotypical people with gender diverse identities	3	23
Newell et al. (2023)	Calculate the prevalence of suicidality in autistic individuals without intellectual disability and review the quality of the research	No	(ASC or ASD or Asperg* or Autis* or ‘high#functioning’ or ‘pervasive developmental disorder’ or PDD or HFA) AND (’possib* autis*’ or ‘autis* trait*’ or ‘autis* phenotyp*’ or ‘undiagnosed autis*’ or ‘self-diagnos* autis*’) AND (suicid* or ‘suicide plans’ or ‘suicide attempts’ or ‘attempted suicide’ or parasuicide ‘self-harm’ or ‘self-inj*’)	Quantitative studies with prevalence estimates of suicidality among autistic individuals without intellectual disability	3	40
Valentine et al. (2024)	Describe research on wait list interventions (i.e., intervention used to support youth and families while the youth waited for mental health assessments and/or interventions)	No	(“wait*”, wait* adj5 initiative, wait* intervention, wait* adj time, Wait* adj5 length, Wait* adj5 duration, Access adj5 delay, Wait* adj5 access) AND (psych*, behav*, CAMHS, Child*, “mental health” or depress*)	Quantitative or qualitative research on youth in interventions while on waitlists for obtaining mental health care	0	18
Wu et al. (2023)	Investigate associations between psychiatric comorbidities with anxiety in individuals with ADHD, autism, or ADHD + Autism	No	Not Reported- “key terms such as “Anxiety,” “ADHD,” and “ASD”	Quantitative and qualitative research on psychiatric comorbidities for those with ADHD, autism, or both	0	8
Current review	Systematic review of all articles related to the intersection of gender diversity and autism	Yes	Transsex* OR transgender* OR gender dysphori*OR gender identity disorder* OR gender identit* OR sexual identit* OR gender diver* OR nonbinary OR non-binary OR gender vari* OR gender nonconform* OR gender non-conform* OR sex reassign* OR transma* OR transfem* OR gender var* OR gender incongruen*AND Pervasive development* disorder* OR pdd OR pdd-nos OR pervasive developmental disorder not otherwise specified OR autis* OR Autism Spectrum Disorder* OR Asperger* OR ASD OR neurodiver* OR autism spectrum* OR autis* trait* OR autism spectrum condition*	Quantitative and qualitative studies on gender diversity and autism	N/A	99 (after August 2018) 120 total

**Table 2:** Quantitative studies included

References	Method and study design	Main focus	Total participants	Biological sex	Gender identity	Age group in focus	Participant country
Aldridge et al. (2021)	Longitudinal	Investigate the impact of gender-affirming hormone therapy on depression and anxiety among transgender individuals and to examine the impact of demographic and psychological factors (e.g., autistic) on changes in anxiety and depression	Total N = 178 (ASD = NR; GD = 178)	Total Male = 95 (ASD = NR; GD = 95), Total Female = 83 (ASD = NR; GD = 83)	NR	17 to 79 years	UK
Arnold et al. (2023)	Case–Control Study	Identify barriers to healthcare and correlates of those barriers for autistic and non-autistic adults in Australia	Total N = 333 (ASD = 263; GD = 70)	NR	Total Male = 107 (ASD = 91; GD = NR), Total Female = 206 (ASD = 151; GD = NR), Total Gender-diverse (non-binary, transgender, intersex) = 21 (ASD = 21; GD = 21)	25 years and older	Australia
Barnett et al. (2021)	Survey	Analyze the association among autism traits and disordered eating controlling for anxiety and depression and moderated by sex/gender	Total N = 686 (ASD = 40; GD = NR)	Total Male = 267 (ASD = NR; GD = NR), Total Female = 419 (ASD = NR; GD = NR)	Total Male = 267 (ASD = NR; GD = NR), Total Female = 419 (ASD = NR; GD = NR)	18 to 70 years	NR (online survey) and UK
Brandsma et al. (2022)	Pre-post intervention study	Look at psychological well-being and complaints, self-esteem, social responsiveness, and gender diversity feelings before and after a group intervention for autistic, gender diverse individuals	Total N = 41 (ASD = 41; GD = 41)	Total Male = 23 (ASD = 23; GD = 23), Total Female = 18 (ASD = 18; GD = 18)	NR	12 to 23 years at T1	Netherlands
Bretherton et al. (2021)	Community-based survey	To identify the mental/physical health conditions, barriers/access to health care, and community views on funding among transgender Australians	Total N = 928 (ASD = 137; GD = 928)	Total Male = 403 (ASD = NR; GD = 403), Total Female = 520 (ASD = NR; GD = 520), Total intersex = 5 (ASD = NR; GD = 5)	Total Male = 91 (ASD = NR; GD = 91), Total Female = 15 (ASD = NR; GD = 15), Total transmale = 239 (ASD = NR; GD = 239), Total transfemale = 202 (ASD = NR; GD = 202), Total nonbinary = 133 (ASD = NR; GD = 133), Total gender queer = 41 (ASD = NR; GD = 41), Total gender neutral = 11 (ASD = NR; GD = 11), Total gender fluid = 19 (ASD = NR; GD = 19), Total intersex = 2 (ASD = NR; GD = 2), Total agender = 20 (ASD = NR; GD = 20), Total other = 30 (ASD = NR; GD = 30)	18 years and older	Australia
Brunissen et al. (2021)	Case–control study	Examine sex differences in gender expression and identity among autistic youth	Total N = 163 (ASD = 163; GD = 32)	Total Male = 119 (ASD = 119: GD = 17), Total Female = 44 (ASD = 44; GD = 15)	Total Male = 114 (ASD = 114; GD = 5), Total Female = 54 (ASD = 54; GD = 10); Total Genderqueer or Gender non-conforming = 17 (ASD = 17; GD = 17)	6 to 21 years	USA
Bush (2019)	Case–control study	Compare sexual desire, behaviors, awareness, and satisfaction among autistic and non-autistic females, and to examine correlations among these measures of sexuality	Total N = 427 (ASD = 248; GD = 130)	Total Female = 427 (ASD = 248; GD = 163)	Total Female = 286 (ASD = 142; GD = 0), Total Agender = 53 (ASD = 42; GD = 53), Total Genderqueer or Non-binary = 50 (ASD = 37; GD = 50), Total Not Entirely Feminine = 23 (ASD = 17; GD = 23), Total Genderfluid = 15 (ASD = 10; GD = 15)	18 to 30 years	International (online survey)
Bush et al. (2021)	Case–control study	Compare sexual desire, behaviors, and anxiety between autistic women with asexual identities and other sexual orientations	Total N = 247 (ASD = 247; GD = 121)	Total Female = 247 (ASD = 247; GD = 121)	Total Female = 126 (ASD = 125; GD = 0), Total Agender = 42 (ASD = 42; GD = 42), Total Genderqueer or non-binary = 37 (ASD = 37; GD = 37), Total demigirl = 17 (ASD = 17; GD = 17), Total Genderfluid = 10 (ASD = 10; GD = 10)	18 to 30 years	International (online survey)
Butler et al. (2019)	Case–control study	Analyze rates of self-harm ideation by self and peer report, bullying, depression, and support in school-aged youth who identify as trans, other gender identity, or cisgender	Total N = 8440 (ASD = NR; GD = 282)	NR	Total Male = 3625 (ASD = NR; GD = 0), Total Female = 4361 (ASD = NR; GD = 0), Total Trans = 55 (ASD = NR; GD = 55), Total Other = 227 (ASD = NR; GD = 227)	13 to 17 years	UK
Camilleri et al. (2024)	Survey	Investigate predictors and correlates of audiences enjoying, comprehending, and succeeding with social stories in the Stories Online for Autism (SOFA) digital application	Aim 1: Total N = 568 (ASD = 461; GD = NR); Aim 2: Total N = 127 (ASD = 102; GD = NR); Aim 3: Total N = 161 (ASD = 127, GD = NR)	NR	Aim 1: Total Male = 401 (ASD = NR; GD = NR), Total Female = 135 (ASD = NR; GD = NR), Total Other = 32 (ASD = NR; GD = 32); Aim 2: Total Male = 92 (ASD = NR; GD = NR), Total Female = 24 (ASD = NR; GD = NR), Total Other = 11 (ASD = NR; GD = 11); Aim 3: Total Male = 11 (ASD = NR; GD = NR); Total Female = 33 (ASD = NR; GD = NR), Total Other = 17 (ASD = NR; GD = 17)	0 to 15 years	International (online survey)
Chang et al. (2022)	Case–control study	Investigate childhood/adolescence predictors for the endorsement of a "wish to be of the opposite sex" in adulthood	Total N = 130 (ASD = 88, GD = 28)	Total Male = 114 (ASD = 79; GD = 22), Total Female = 16 (ASD = 9; GD = 6)	Total Male = 98 (ASD = 65; GD = 6), Total Female = 32 (ASD = 23; GD = 22)	6.5 to 19.3 years	Taiwan
Chao et al. (2023)	Clinical chart review	Establish the prevalence of GD diagnoses and co-occurring conditions in Taiwan between the years 2010 to 2019	Total N = 23,500,000 (ASD = 30; GD = 4940)	Total Male = NR (ASD = 26; GD = 3680), Total Female = NR (ASD = 3; GD = 1260)	NR	0 to ≥ 18 years	Taiwan
Cheung et al. (2018)	Cohort and clinical chart review	Determine the prevalence of autism and ADHD in referrals to a transgender clinic, and describe the sociodemographic characteristics of the sample	Total N = 540 (ASD = 26; GD = 540)	NR	Total Male = 238 (ASD = NR; GD = 238), Total Female = 196 (ASD = NR; GD = 196), Total Nonbinary = 99 (ASD = NR; GD = 99), Total Unassigned = 7 (ASD = NR; GD = 7)	14 to 17 years	Australia
Clyde et al. (2024)	Survey	Identify the SCQ cut-off score for transgender autistic youth, identify the correspondence between ASD diagnoses and positive SCQ screens, determine the relation between parent- and youth-reported anxiety symptoms and ASD traits	Youth = 325 (ASD = 17, Possible ASD = 5; GD = 325); Parents = 553 (ASD = NR; GD = NR)	Total Male = 115 (ASD = NR; GD = 115), Total Female = 210 (ASD = NR; GD = 210)	Total Male = 182 (ASD = NR; GD = 182), Total Masculine Spectrum = 25 (ASD = NR; GD = 25), Total Female = 100 (ASD = NR; GD = 100), Total Feminine Spectrum = 7 (ASD = NR; GD = 7), Non-binary/agender = 6 (ASD = NR; GD = 6), Unsure = 2 (ASD = NR; GD = 6)	7.4 to 18.2 years	USA
Coburn and Williams (2022)	Case–control study	Explore communication trait differences between cisgender and gender diverse autistic adults	Total N = 20 (ASD = 15, GD = 15)	Total Male = 6 (ASD = 5; GD = 1), Total Female = 14 (ASD = 10; GD = 14)	Total Male = 8 (ASD = 6, GD = 3), Total Female = 7 (ASD = 4, GD = 7), Total Nonbinary = 5 (ASD = 5, GD = 5)	20 to 57 years	NR (online)
Corbett et al. (2023)	Case–control study	Investigate self-reported binary and non-binary gender experiences in autistic youth, examine consistency between parent-report and youth self-report of gender diversity experiences, and consider the relations among parent- and youth-reported gender diversity with internalizing symptoms	Youth = 244 (ASD = 140; GD = 5)	Youth Male = 162 (ASD = 104; GD = NR), Youth Female = 82 (ASD = 36; GD = NR)	NR	10.5 to 12.92 years	USA
David et al. (2023)	Clinical chart review and case–control study	Present the prevalence of autism diagnoses in Norwegian adolescents referred for gender-affirming care, represent autism traits in that population, and investigate autism trait differences based on sex assigned at birth or the presence/absence of a gender diverse identity	Total N = 83 (ASD = 8; GD = 83); SRS normative sample	Total Male = 21 (ASD = 0; GD = 21), Total Female = 62 (ASD = 8; GD = 62)	NR	13 to 18 years	Netherlands
Ghassabian et al. (2022)	Case–control, cohort, and longitudinal study	Present the prevalence and stability of gender diversity in a population-based cohort of youth and adolescents and examine mental health correlates	Total N = 5727 (ASD = NR; GD = 210)	T1: Total Male = 2450 (ASD = NR; GD = 20); Total Female = 2466 (ASD = NR; GD = 11) T2: Total Male = 2316 (ASD = NR; GD = 7); Total Female = 2346 (ASD = NR; GD = 13)	NR	9 to 11 years and 13 to 15 years	Netherlands
Greenspan et al. (2023)	Survey	Identify school and community protective factors for gender diverse, autistic youth	Total N = 31 (ASD = 20; GD = 24)	NR	Total Cisgender = 7 (ASD = NR; GD = 0), Total Transgender = 7 (ASD = NR; GD = 7), Total Non-binary = 13 (ASD = NR; GD = 13), Total Agender = 2 (ASD = NR; GD = 2), Total Questioning = 2 (ASD = NR; GD = 2)	13 to 17 years	International (Online Survey)
Hall et al. (2020)	Case–control study and qualitative interviews	Investigate health and healthcare of autistic individuals who identify as LGBTQ +	Total N = 54 (ASD = 54; GD = NR)	NR	Total Male = 26 (ASD = 26; GD = NR), Total Female = 21 (ASD = 21; GD = NR), Other = 7 (ASD = 7; GD = NR)	18 to 58 years	USA
Hendricks et al. (2022)	Case–control study	Examine the association of autism traits with gender diversity from the perspective of the extreme male brain theory	Total N = 89 (ASD = 6; GD = 50)	Total Male = 36 (ASD = 7; GD = 18), Total Female = 53 (ASD = 30; GD = 32)	Total Male = 46 (ASD = 22; GD = 32), Total Female = 39 (ASD = 15; GD = 18)	18 years and older	UK
Hermann et al. (2021)	Clinical chart review	Analyze rates of ASD in a German Gender Diversity Clinic, and present other characteristics of individuals with dual diagnoses	Total N = 579 (ASD = 18; GD = 58)	Total Male = 136 (ASD = 7; GD = 136), Total Female = 443 (ASD = 11; GD = 443)	NR	10 years and older	Germany
Hill et al. (2020)	Case–control study and clinical chart review	Present the clinical characteristics of patients with gender diverse identities admitted to a mixed gender secure psychiatric hospital for adolescents	Total N = 41 (ASD = NR, GD = 13)	Total Male = 0, Total Female = 41 (ASD = NR, GD = 13)	Total Male = 6 (ASD = NR, GD = 6), Total Female = 28 (ASD = NR, GD = 0), Total Other = 7 (ASD = NR, GD = 7),	13.8 to 17.8 years	UK
Hilton et al. (2022)	Survey	Examine the frequency of ASD traits in youth presenting to a hospital-based gender clinic, and the impact of ASD traits on distress related to gender	Total N = 219 (ASD = 4; GD = 64)	Total Male = 75 (ASD = NR; GD = 24), Total Female = 144 (ASD = NR; GD = 40)	Total Male = 91 (ASD = NR; GD = 40), Total Female = 128 (ASD = NR; GD = 24)	8 to 16 years	Australia
Hisle-Gorman et al. (2019)	Case–control study	Investigate an overrepresentation of GD in autistic children	Total N = 292,572 (ASD = 48,762; GD = 66)	Total Male = 234,058 (ASD = 39,010; GD = 52), Total Female = 58,514 (ASD = 9752; GD = 14)	NR	2 to 18 years	USA
Hull et al. (2019)	Case–control stuey	Test gender and diagnostic differences in self-reported camouflaging and consider whether gender differences remain when account for autism traits	Total N = 778 (ASD = 306; GD = NR)	NR	Total Male = 301 (ASD = 108, GD = NR), Total Female = 434 (ASD = 182, GD = NR), Total Non-Binary = 43 (ASD = 16, GD = 43)	15 years and older	UK
Hull et al. (2021)	Case–control study	Examine the association among camouflaging with anxiety and depression controlling for autistic traits, consider gender as a moderator in the relation between camouflaging and social anxiety, generalized anxiety, and depression, and assess the risk of mental health problems at different levels of camouflaging	Total N = 305 (ASD = 305, GD = 22)	NR	Total Male = 104 (ASD = 104; GD = NR), Total Female = 181 (ASD = 181, GD = NR), Total Ninbinary = 18 (ASD = 18, GD = 18)	18 to 75 years	UK
Kahraman et al. (2021)	Case–control study	Compare youth with and without gender diverse identities in empathy, emotional recognition, and social skills	Total N = 36 (ASD = 0; GD = 17)	Total Male = 15 (ASD = 0; GD = 6), Total Female = 21 (ASD = 0; GD = 11)	NR	13 to 18 years	Turkey
Kallitsounaki and Williams (2020a, 2020b) (Mentalising Moderates the Link between Autism Traits and Current Gender Dysphoric Features in Primarily Non-autistic, Cisgender Individuals)	Survey	Investigate the nature of the association among autism, GD, and mentalising	Total N = 101 (ASD = 13; GD = 0)	Total Male = 51 (ASD = NR; GD = 0), Total Female = 50 (ASD = NR; GD = 0)	Total Male = 51 (ASD = NR; GD = NR), Total Female = 50 (ASD = NR; GD = NR)	22 to 70 years	International (online survey)
Kallitsounaki and Williams (2020a, 2020b) (A Relation Between Autism Traits and Gender Self-concept: Evidence from Explicit and Implicit measures)	Survey and case–control study	Examine the link among autism traits with explicit and implicit gender self-concept in the general population	Total N = 101 (ASD = 13; GD = 0)	Total Male = 51 (ASD = NR; GD = 0), Total Female = 50 (ASD = NR; GD = 0)	Total Male = 51 (ASD = NR; GD = NR), Total Female = 50 (ASD = NR; GD = NR)	22 to 70 years	International (online survey)
Kallitsounaki et al. (2021)	Survey	Replicate Kallitsounaki and Williams findings of a link among autism traits, gender diversity, recalled cross gender behavior, and mentalising in adults from the general population and to assess mentalising ability as a mediator in the relation among autism traits and gender diversity feelings	Total N = 126 (ASD = 2; GD = NR)	Total Male = 29 (ASD = NR; GD = NR), Total Female = 97 (ASD = NR; GD = NR)	NR	18 to 45 years	International (online survey)
Kallitsounaki and Williams (2022a, 2022b)	Case–control study	Investigate the impact of ASD traits on gender-related cognition, if autistic people have higher rates of gender diversity and recall less gender-typed behavior in childhood, and if transgender indiviuals have elevated ASD traits	Total N = 347 (ASD = 163; GD = 134)	Total Male = 171 (ASD = 79; GD = 66), Total Female = 176 (ASD = 84; GD = 68)	Total Male = 173 (ASD 77; GD = 66), Total Female = 174 (ASD = 86; GD = 98)	18 to 70 years	International (online survey)
Kallitsounaki and Williams (2023)	Case–control study	Examine alexithymia in autistic and non-autistic transgender adults	Total N = 347 (ASD = 163; GD = 134)	Total Male = 171 (ASD = 79; GD = 66), Total Female = 176 (ASD = 84; GD = 68)	Total Male = 173 (ASD 77; GD = 66), Total Female = 174 (ASD = 86; GD = 98)	18 to 70 years	International (online survey)
Kaltiala-Heino et al. (2019)	Clinical chart review	Sexual experiences of clinically referred adolescents with features of gender dysphoria	Total N = 182,798 (In GD population, Autistic = 17; GD = 101)	Total Male = 90,968 (ASD = NR; GD = NR); Subsample Total Male = 15 (ASD = 2; GD = 15), Total Female = 91,830 (ASD = NR; GD = 84); Subsample Total Female = 84 (ASD = 15)	NR	14 to 18 years	Finland
Khan et al. (2023a, 2023b) (Co-occurring Gender Dysphoria and Autism Spectrum Disorder in Adolescents)	Case–control study	Examine the prevalence of co-occurring autism and gender diversity among youth and identify demographic differences in diagnosis of gender dysphoria among autistic and non-autistic youth	Total N = 919,898 (ASD = 40,713; GD = 5389)	Total Male = 467,365 (ASD = 30,631; GD = 1407), Total Female = 452,503 (ASD = 10,082; GD = 3982)	NR	9 to 18 years	USA
Khan et al. (2023a, 2023b) (Mental Health of Youth with Autism Spectrum Disorder and Gender Dysphoria)	Case–control study	Understand the associations among autism, gender diversity, and mental health diagnoses in a large sample of adolescents in the US	Total N = 919, 868 (ASD = 40,713; GD = 5389)	Total Male = 467,365 (ASD = NR; GD = NR), Total Female = 452,503 (ASD = NR; GD = NR)	NR	9 to 18 years	USA
Koffer Miller et al. (2022)	Cohort and survey study	Identify service needs and barriers to those needs for autistic individuals with gender diversity, as well as gender differences in those needs and barriers	Total N = 1204 (ASD = 1204; GD = 36)	Total Male = 748 (ASD = 748; GD = 14), Total Female = 293 (ASD = 293; GD = 20)	Total Male = 847 (ASD = 847; GD = 3), Total Female = 327 (ASD = 327; GD = 3); Total Other Gender = 30 (ASD = 30; GD = 30)	18 years and older	USA
Kung (2020)	Case–control study	Examine traits related to autism, the extreme male brain theory, and the mind blindness theory in transgender and non-binary adults	Total N = 323 (ASD = 0; GD = 323)	Total Male = 145 (ASD = NR; GD = 145), Total Female = 178 (ASD = NR; GD = 178)	Total Male = 74 (ASD = NR; GD = 74), Total Female = 95 (ASD = NR; GD = 95), Total Nonbinary = 154 (ASD = NR; GD = 154)	18 to 76 years	International (USA and UK)
Kung (2023)	Case–control study	Investigate the associations among autistic traits, gender minority stress, and mental health among transgender and non-binary adults	Total N = 308 (ASD = NR; GD = 308)	Total Male = 138 (ASD = NR; GD = 138), Total Female = 170 (ASD = NR; GD = 170)	Total Male = 72 (ASD = NR; GD = 72), Total Female = 90 (ASD = NR; GD = 90), Total Non-Binary = 146 (ASD = NR; GD = 146)	18 to 76 years	International (USA and UK)
Leef et al. (2019)	Case–control and clinical chart review study	Compare autism in school-aged children referred for GD to school-aged children referred for other clinical concerns	Total N = 101 (ASD = 13; GD = 61)	Total Male = 73 (ASD = 12; GD = 45), Total Female = 28 (ASD = 1; GD = 16)	NR	4 to 13 years	Canada
Lehmann et al. (2020)	Survey	Determine the prevalence of autism traits in adults with GD	Total N = 123 (ASD = NR; GD = 123)	Total Male = 57 (ASD = NR; GD = 57), Total Female = 66 (ASD = NR; GD = 66)	Total Male = 42 (ASD = NR; GD = 42), Total Female = 33 (ASD = NR; GD = 33), Total Transmale/transfemale = 42 (ASD = NR; GD = 42), Total Non-binary = 6 (ASD = NR; GD = 6)	16 years and older	Northern Ireland
Mahfouda et al. (2019)	Cohort, clinical chart review, and case–control study	Analyze rates of ASD and psychopathology in gender diverse youth	Total N = 104 (ASD = 23; GD = 104)	Total Male = 71 (ASD = 4; GD = 71), Total Female = 79 (ASD = 19; GD = 79)	Total Male = 71 (ASD = 15; GD = 71), Total Female = 23 (ASD = 4; GD = 23), Total Nonbinary = 6 (ASD = 2; GD = 6), Total Not Specified = 4 (ASD = 2; GD = 2)	18 years and younger	Australia
Mazzoli et al. (2022)	Case–control, chart review, and longitudinal study	Evaluate differences in AQ score between hormone-naïve transgender people and cisgender people, the impact of gender-affirming hormonal treatment on AQ scores, and alexithymia and social anxiety as mediators of change in AQ scores	Cross-sectional study: Total N = 789 (ASD = NR; GD = 388); Longitudinal study: Total N = 62 (ASD = NR; GD = 62)	Cross-sectional study: Total Male = 388 (ASD = NR; GD = 182), Total Female = 378 (ASD = NR; GD = 206); Longitudinal study: Total Male = 24 (ASD = NR; GD = 24), Total Female = 38 (ASD = NR; GD = 38)	Cross-sectional study: Total Male = 435 (ASD = NR; GD = 206), Total Female = 354 (ASD = NR; GD = 182); Longitudinal study: Total Male = 38 (ASD = NR; GD = 38), Total Female = 24 (ASD = NR; GD = 24)	18 years and older	Italy
McLellan et al. (2023)	Case–control study	Investigate how autism-related differences may impact the self-report of stigmatization in transgender youth and consider the impact of cognitive and developmental factors	Total N = 65 (ASD = NR; GD = 65)	Total Male = 37 (ASD = NR; GD = 37), Total Female = 28 (ASD = NR; GD = 28)	Total Male = 28 (ASD = NR; GD = 28), Total Female = 37 (ASD = NR; GD = 37)	13 to 21 years	USA
McPhate et al. (2021)	Case–control and clinical chart review study	Assess rates of gender diversity in youth with neurodevelopmental and psychiatric conditions	Total N = 4944 (ASD = NR; GD = 128)	Total Male = 2944 (ASD = NR; GD = 55), Total Female = 2000 (ASD = NR, GD = 73)	Total Male = 2962 (ASD = NR; GD = 73), Total Female = 1982 (ASD = NR; GD = 55)	6 to 18 years	Australia
McQuaid et al. (2023)	Survey	Examine the effects of sex, gender identity, and diagnostic timing on camouflaging in autistic adults	Total N = 502 (ASD = 502; GD = 62)	Total Male = 226 (ASD = 226; GD = NR), Total Female = 276 (ASD = 276; GD = NR)	Total gender diverse = 62 (ASD = 62), Total cisgender = 440 (ASD = 440)	18 to 49 years	International (online survey)
McQuaid et al., (2023a, 2023b, 2023c)	Case–control study	Explore differences in mental health and subjective quality of life between "sexual minority" and heterosexual adults	Total N = 651 (ASD = 651; GD = 67)	Total Male = 258 (ASD = 258; GD = NR), Total Female = 393 (ASD = 393; GD = NR)	NR	18.5 to 83.33 years	USA
Munoz Murakami et al. (2022)	Survey	Investigate the relation among the CBCL item regarding ASD and gender diversity	Total N = 1719 (ASD = 0; GD = 11)	Total Male = 839 (ASD = 0, GD = NR), Total Female = 880 (ASD = 0, GD = NR)	Same gender as sex assigned at birth = 1259 (ASD = 0; GD = 0), Different from sex assigned at birth = 11 (ASD = 0; GD = 11)	6 to 12 years	Canada
Murphy et al. (2020)	Case–control and survey study	Investigate the overlap of ASD and gender diversity, and the effect of the intersection on depression and anxiety	Total N = 727 (ASD = 62; GD = 124)	NR	Total Male = 188 (ASD = 31; GD = 76), Total Female = 539 (ASD = 32; GD = 48)	18 to 74 years	International (online survey)
Nabbijohn et al. (2019)	Case–control and survey study	Examine the association among gender variance and autism spectrum disorder among children with and without mental health diagnoses	Total N = 2445 (ASD = 80; GD = NR)	Total Male = 1258 (ASD = 57; GD = NR), Total Female = 1187 (ASD = 23; GD = NR)	NR	6 to 12 years	International
Nobili et al. (2020)	Longitudinal	Explore the autism traits over time in transgender people after cross-sex hormone treatment, controlling for changes in anxiety and age and to explore the impact of sex assigned at birth and changes in anxiety on changes in autism traits over time	Total N = 118 (ASD = 0; GD = 118)	Total Male = 59 (ASD = NR; GD = 59), Total Female = 59 (ASD = NR; GD = 59)	Total Male = 59 (ASD = NR; GD = 59), Total Female = 59 (ASD = NR; GD = 59)	14 to 41 years	UK
Nunes-Moreno et al. (2022)	Cohort, clinical chart review, and case control study	Assess the odds of a psychiatric or neurodevelopmental diagnosis in youth with or without a gender dysphoria diagnosis	Total N = 20,821 (ASD = 653; GD = 4173)	Total Male = 6925 (ASD = NR; GD = 2407), Total Female = 13,896 (ASD = NR; GD = 2766)	NR	3.4 to 28.5 years	USA
Pecora et al. (2020)	Case–control and cohort study	Investigate gender and sexual diversity among autistic females, as well as rates of regretted, unwanted, and sexual encounters among females who identify as transgender and non-heterosexual	Total N = 284 (ASD = 123; GD = 40)	Total Male = 0 (ASD = 0; GD = 0), Total Female = 284 (ASD = 123; GD = 40)	NB: 11 individuals were excluded from the following sample: Total Male = 6 (ASD = 4; GD = 6), Total Female = 255 (ASD = 108; GD = 255), Total Other = 34 (ASD = 22; GD = 34)	18 to 56 years	International
Ristori et al. (2020)	Longitudinal and cross-sectional	Examine sexual distress and its psychological and biological correlates in transgender individuals who have not undergone surgery, and determine the effect of hormonal treatment on sexual distress in this population	Time point 1: Total N = 301 (ASD = NR; GD = 301), Time point 2: Total N = 72 (ASD = NR; GD = 72)	Time point 1: Total Male = 160 (ASD = NR; GD = 160), Total Female = 141 (ASD = NR; GD = 141); Time point 2: Total Male = 38 (ASD = NR), Total Female = 40 (ASD = NR)	Time point 1: Total Male = 141 (ASD = NR; GD = 141);, Total Female = 160 (ASD = NR; GD = 160); Time point 2: Total Male = 40 (ASD = NR; GD = 40), Total Female = 38 (ASD = NR; GD = 38)	18 years and older	Italy
Russell et al. (2021)	Longitudinal study	Examine changes over time in SRS-2 scores in clinic referred, gender diverse youth	Total N = 95 (ASD = NR; GD = 95)	Total Male = 38 (ASD = NR; GD = 38), Total Female = 57 (ASD = NR; GD = 57)	NR	T1: 9.9 to 15.9 years, T2: 10.9 to 16.6 years	UK
Saunders et al. (2023)	Case control clinical chart review study	Describe the demographic characteristics, health conditions, and healthcare experiences of trans and non-binary adults in England	Total N = 840,691 (ASD = NR; GD = 6333); Among those with long-term health outcome data: Total N = 735,078 (ASD = 4463, GD = 5110)	NR	Total Male = 361,237 (ASD = NR; GD = 1971), Total Female = 470,666 (ASD = NR; GD = 1708), Total Non-binary = 1220 (ASD = NR; GD = 1220), Total Prefer to Self-Describe = 1047 (ASD = NR, GD = 1047), Total Prefer Not to Say = 103 (ASD = NR, GD = 103)	16 to 84 years	UK
Schlitz et al. (2021)	Case–control study	Examine the relations among the broader autism phenotype, gender nonconformity and internalizing symptoms	Total N = 174 (ASD = NR; GD = NR)	Total Male = 49 (ASD = NR; GD = NR), Total Female = 125 (ASD = NR; GD = NR)	Total Male = 48 (ASD = NR; GD = NR), Total Female = 125 (ASD = NR; GD = NR), Total Intersex = 1 (ASD = NR; GD = NR)	18 to 22 years	USA
Stagg and Vincent (2019)	Case–control and survey study	Compare autism traits among individuals who identify as cisgender, transgender, and nonbinary	Total N = 177 (ASD = 18; GD = 109)	Total Male = 66 (ASD = 7; GD = 38), Total Female = 111 (ASD = 11; GD = 50)	Total Male = 59 (ASD = 4; GD = 31), Total Female = 59 (ASD = 1; GD = 19), Total Nonbinary = 59 (ASD = 13; GD = 59)	18 years and older	UK
Steinberg et al. (2022)	Case–control study	Demonstrate the need for improved measures of gender identity for autistic adults by presenting the accuracy of a standard question about sex and gender	Total N = 1527 (ASD = 1527; GD = 35 to 60, depending on the measure)	Total Male = 1031 (ASD = 1031; GD = 18); Total Female = 436 (ASD = 436; GD = 24)	Total Male = 1015 (ASD = 1015; GD = 27), Total Female = 418 (ASD = 418; GD = 15), Total Other = 45 (ASD = 35; GD = 34)	NR (adults)	USA
Strang et al. (2022)	Case–control study	Investigate mental health in autistic-transgender, non-autistic transgender, and autistic-cisgender adolescents	Total N = 120 (ASD = 94; GD = 93)	Total Male = 60 (ASD = 53; GD = 44), Total Female = 60 (ASD = 41; GD = 49)	Total Male = 55 (ASD = 36; GD = 36), Total Female = 65 (ASD = 58; GD = 58)	13 to 21 years	USA
Strang et al. (2022)	Case–control study	Examine the relations among executive functioning with an autism spectrum disorder diagnosis, internalizing symptoms, and gender-affirming medical intervention status	Total N = 124 (ASD = 35; GD = 124)	Total Male = 42 (ASD = NR; GD = 42), Total Female = 82 (ASD = NR; GD = 82)	Total Male = 81 (ASD = NR; GD = 81), Total female = 41 (ASD = NR; GD = 41), Total Nonbinary = 2 (ASD = NR; GD = 2)	11 to 21 years	USA
Strang et al., (2023a, 2023b, 2023c, 2023d)	Case–control study	In a sample of non-autistic, slightly sub clinically autistic, and diagnosed autistic individuals, examine: default mode neural functional connectivity, consider how default mode functional connectivity relates to autism traits, internalizing psychopathology, gender dysphoria, and perceived sexual/gender-minority related stigma, and investigate gender-related default mode neural functional connectivity	Total N = 45 (ASD = 15; Subclinical ASD = 14; GD = 45)	NR	Total Male = 26 (ASD = 9; GD = 26), Total Female = 19 (ASD = 9; GD = 19)	13 to 21 years	USA
Strang et al., (2023a, 2023b, 2023c, 2023d)	Psychometric analysis of the Gender Self-Report	Calibrate and validate the Gender Self-Report	Study 1 Calibration: Total N = 1654 (ASD = 621; GD = 600); Study 2 Validation: Total N = 1442 (ASD = NR; GD = 385)	Study 1 Calibration: Total Male = 431 (ASD = 197; GD = NR), Total Female = 1222 (ASD = 423; GD = NR); Study 2 Validation: NR	Study 1 Calibration: Total Transgender = 243 (ASD = NR; GD = 243), Total Binary Cisgender = 1054 (ASD = NR; GD = 0), Total Nonbinary = 142 (ASD = NR; GD = 142), Total Questioning = 51 (ASD = NR; GD = 51), Total Fluid = 68 (ASD = NR; GD = 68), Total Genderqueer = 82 (ASD = NR; GD = 82), Total Agender = 41 (ASD = NR; GD = 41), Total Demigender = 65 (ASD = NR; GD = 65), Total Third Gender = 33 (ASD = NR; GD = 33), Total Unreported = 58 (ASD = NR; GD = NR); Study 2 Validation: Total Cisgender = 1057 (ASD = NR; GD = 1057), Total Nonbinary = 142 (ASD = NR; GD = 142), Total Binary Transgender = 243 (ASD = NR; GD = 243)	Study 1 Calibration: 10 to 77.25 years; Stud 2 Validation: NR	USA
Strang et al., (2023a, 2023b, 2023c, 2023d)	Delphi and community based participatory research	Develop and refine the first self-report/self-advocacy tool for autistic gender-diverse young adults that focuses on needs, risks, and resilience using Delphi and community-based approaches	Study Personnel: Total N = 8 (ASD = 5; GD = 6); Experts: Total N = 25 (ASD = 16; GD = 16)	NR	Study Personnel: Total Male = 3 (ASD = NR; GD = NR), Total Female = 4 (ASD = NR; GD = NR), Total Gender-Diverse = 3 (ASD = NR; GD = 3), Total Gender-Exploring/Expansive/Queer = 2 (ASD = NR; GD = 2), Total Agender = 1 (ASD = NR; GD = 1); Experts: Total Cisgender Male = 1 (ASD = 0; GD = 0), Total Cisgender Female = 7 (ASD = 0; GD = 0), Total Femme nonconforming = 1 (ASD = 0; GD = 1), Total Formerly Transgender Male current Tomboy Female = 1 (ASD = 1; GD = 1), Total Transgender Male/Transmasculine = 2 (ASD = 2; GD = 2), Total Transgender Female/Transfeminine = 5 (ASD = 5; GD = 5), Total Genderqueer = 1 (ASD = 1; GD = 1), Total Nonbinary = 7 (ASD = 7; GD = 7)	21 to 68 years	International (USA and Netherlands)
Strauss et al. (2021)	Survey study	Analyze the prevalence of autism, the rate of mental health problems, and experiences accessing gender-affirming care in trans youth	Total N = 859 (ASD = 172; GD = 859)	NR	NR	14 to 25 years	Australia
Sumia and Kalitala (2021)	Case–control study	Describe the co-occurring psychiatric disorders, age at onset of gender diverse identity, pubertal timing, as well as peer and romantic relationships among gender-referred autistic and non-autistic adolescents	Total N = 106 (ASD = 19, GD = 106)	Total Male = 53 (ASD = NR; GD = 53), Total Female = 53 (ASD = NR; GD = 53)	Total Male = 53 (ASD = NR; GD = 53), Total Female = 53 (ASD = NR; GD = 53)	15 to 18 years	Finland
Tikkinen et al. (2019)	Population-based survey	Assess the mental health problems that are perceived as diseases by medical professionals involved and uninvolved in psychiatry (physicians, nurses), government officials, and the general public	Total N = 3280 (ASD = NR; GD = NR)	NR	NR	General public: 18 to 75 years, Medical professionals: under 65 years	Finland
Tollit et al. (2021)	Clinical Chart Review	Illuminate the clinical profile of transgender and gender diverse patients in an Australian clinic	Total N = 359 (ASD = 58; GD = 3)	Total Male = 166 (ASD = 37; GD = 162), Total Female = 193 (ASD = 21; GD = 191)	Total Male = 178 (ASD = NR; GD = 174), Total Female = 141 (ASD = NR; GD = 139), Total Non-Binary = 26 (ASD = NR; GD = 26), Total Not Sure = 14 (ASD = NR; GD = 14)	3.6 to 18.1 years	Australia
van der Miesen et al. (2023)	Case–control study	Assess the association among gender diversity and autism in a sample of Chinese children as well as whether specific subdomains on the Autism Spectrum Quotient and sex-specific factors were associated with gender diversity	Total N = 379 (ASD = 0; GD = 0)	Total Male = 187 (ASD = 0; GD = 0), Total Female = 192 (ASD = 0; GD = 0)	NR	4 to 12 years	Netherlands
Wallisch (2023)	Case–control study	Compare the unmet health care needs between autistic/LGBTQ + individuals and autistic/cisgender/straight individuals and examine the state policy and demographic factors that contribute to those unmet needs	Total N = 120 (ASD = 120; GD = NR)	NR	Total Male = 48 (ASD = 48; GD = NR), Total Female = 46 (ASD = 46; GD = NR), Total Other = 26 (ASD = 26; GD = 26)	18 to 64 years	USA
Warrier et al. (2020)	Case–control and survey study	Compare gender diverse individuals and cisgender individuals on autism diagnoses, autistic traits, suspected autism, and neurodevelopmental disorders and psychiatric conditions associated with autism	Total N = 640,808 (ASD = 27,919; GD = 3777)	NR	Total Male = 252,976 (ASD = NR; GD = NR), Total Female = 384,055 (ASD = NR; GD = NR), Total Transgender/Nonbinary/Other = 3777 (ASD = NR; GD = 3777)	15 to 90 years	International

NR, not reported; GD, gender diverse

### Comparison Between Reviews

#### Comparison with Other Systematic Reviews

A comparison between the current review and all other systematic reviews that were published between 2018 and 2024 and mention one of the keywords is presented in Table 1. Although 18 reviews published between 2018 and 2024 met inclusion criteria, only 9 of those reviews specifically focused on the intersection of autism and gender diversity. There were overlapping studies between the current review and 12 of the recently published review articles, but the current main results identified 59 additional studies that were published after August 2018 that were not captured in previously published reviews. The reason that some reviews did not have overlapping studies with the current review was because they mostly included studies prior to 2018 and did not focus on the intersection, specifically.

### Review of Research Published After 2018

Table 2 presents descriptive statistics on year of publication, type of study design and methods used, the main focus of the article, general characteristics of the participants, and country of origin of study/authors for quantitative studies and Table 3 presents the same information for qualitative studies. Additional information, including journal name, research field, and country of origin of participants is in the supplemental materials both for studies that were published after August 2018 through January 2024 (Supplementary Materials Table S1). Information about studies published during or prior to August 2018 that were not included in the 2018 reviews due to changes in search terms but are presented in Supplementary Materials Table S2.

**Table 3:** Qualitative studies included

References	Method and study design	Main focus	Total participants	Biological sex	Gender identity	Age group in focus	Participant country
Allen-Biddell and Bond (2022)	Qualitative Interview	Describe educational psychologists' experiences and practices with youth who are autistic and gender diverse	Total N = 5 (ASD = NR; GD = NR)	NR	NR	NR	UK
Brilhante (2021)	Qualitative interview	Document the sexuality-related needs of autistic individuals	Total N = 14 (ASD = 14; GD = NR)	NR	Total Male = 8 (ASD = 8; GD = NR), Total Female = 5 (ASD = 5; GD = NR), total Nonbinary = 1 (ASD = 1; GD = 1)	15 to 17 years	Brazil
Cain and Velasco (2021)	Case study	Present a case study of an autistic individual who transitioned from female to male and then identified as non-binary	Total N = 1 (ASD = 1; GD = 1)	Total Female = 1 (ASD = NR; GD = 1)	Total non-binary = 1 (ASD = 1; GD = 1)	NR	International (USA and NR)
Carlile (2020)	Qualitative interview	Describe the experiences of transgender youth and their families in interactions with healthcare providers	Total N = 65 (ASD = children across 27 families; GD = children across 27 families)	NR	NR (all children were transgender or nonbinary)	12 years and older	UK
Coleman-Smith et al. (2020)	Case Study, Grounded Theory	Understand the experience of gender dysphoria for people with autism	Total N = 10 (ASD = 10; GD = 10)	NR	Total Male = 4 (ASD = 4; GD = 4), Total Female = 3 (ASD = 3; GD = 3), Total Non-binary/gender queer = 3 (ASD = 3; GD = 3)	18 to 65 years	UK
Cooper et al. (2022) (Phenomenology of gender dysphoria in autism: A multiperspective qualitative analysis)	Qualitative interview	Generate an understanding of the phenomenology of gender diversity in autistic individuals	Adults = 21 (ASD = 21; GD = 21), Youth = 15 (ASD = 15; GD = 15), Parents = 16 (ASD = NR; GD = NR), Clinicians = 16 (ASD = NR; GD = NR)	Adult Male = 9 (ASD = 9; GD = 9), Adult Female = 12 (ASD = 12; GD = 12); Youth Male = 3 (ASD = 3; GD = 3), Youth Female = 12 (ASD = 12; GD = 12); Parent Sex = NR; Clinician Sex = NR	Adult Male = 7 (ASD = 7; GD = 7), Adult Female = 8 (ASD = 8; GD = 8), Adult Non-binary/Genderqueer = 6 (ASD = 6; GD = 6); Youth Male = 9 (ASD = 9; GD = 9), Youth Female = 3 (ASD = 3; GD = 3), Youth Non-binary/Genderqueer = 3 (ASD = 3; GD = 3); Parent Male = 2 (ASD = NR; GD = NR), Parent Female = 14 (ASD = NR; GD = NR); Clinician Male = 3 (ASD = NR; GD = NR), Clinician Female = 13 (ASD = NR; GD = NR)	Youth: 13 to 17 years; Adults: 18 years and older	UK
Cooper et al. (2023a) (The lived experience of gender dysphoria in autistic adults: An interpretative phenomological analysis)	Qualitative interview	Present the experience of transgender autistic people in their gender identity exploration	Total N = 21 (ASD = 21; GD = 21)	Total Male = 9 (ASD = 9; GD = 9), Total Female = 12 (ASD = 12; GD = 12)	Total Male = 7 (ASD = 7; GD = 7), Total Female = 8 (ASD = 8; GD = 8), Total Nonbinary/genderqueer = 6 (ASD = 6; GD = 6)	18 to 51 years	UK
Cooper et al. (2023b) (Healthcare clinician perspectives on the intersection of autism and gender dysphoria)	Qualitative interview	Illuminate the perspectives of clinicians working with autistic patients with gender diverse identities	Total N = 16 (ASD = NR; GD = NR)	NR	Total Male = 3 (ASD = NR; GD = NR), Total Female = 13 (ASD = NR; GD = NR)	NR	International (online survey)
Folta et al. (2022)	Qualitative interview	Present leisure-time activities for autistic youth as they transition to adulthood	Total N = 18 (ASD = 18; GD = 4)	NR	Total Male = 9 (ASD = 9; GD = 0), Total Female = 5 (ASD = 5; GD = 0), Total Agender/Non-binary = 3 (ASD = 3; GD = 3), Total Transgender = 1 (ASD = 1; GD = 1)	18 to 23 years	USA
Genovese et al. (2023)	Case series	Highlight mental health concerns in gender diverse Autistic individuals	Total N = 4 (ASD = 4; GD = 4)	Total Male = 1 (ASD = 1; GD = 1), Total Female = 3 (ASD = 3; GD = 3)	Total Male = 2 (ASD = 2; GD = 2), Total Female = 1 (ASD = 1; GD = 1), Total Nonbinary = 1 (ASD = 1; GD = 1)	19 to 36 years	USA
Glackin et al. (2023)	Qualitative interview	Describe the gender journeys and experiences of autistic adults, as well as autistic adults' preferences for health care professional treatment	Total N = 12 (ASD = 12, GD = 12)	NR	Total Transmasculine = 3 (ASD = 1, GD = 3), Total Genderqueer Female = 1 (ASD = 1, GD = 1), Total Non-binary Female = 1 (ASD = 1, GD = 1), Total Non-binary Agender = 1 (ASD = 1, GD = 1), Total Genderqueer = 1 (ASD = 1, GD = 1), Total Non-binary = 5 (ASD = 2, GD = 5)	18 years and older	International (online survey)
Grove et al. (2023)	Qualitative interview	Understand the everyday experience of autistic women and gender diverse individuals	Total N = 31 (ASD = 26, GD = 5)	NR	Total Female = 26 (ASD = NR, GD = NR), Total Transgender = 1 (ASD = NR, GD = 1), Total Non-binary = 2 (ASD = NR, GD = 2), Total Autistic gender = 1 (ASD = 1, GD = 1), Total Gender Fluid = 1 (ASD = 1, GD = 1)	21 to 63 years	Australia
Guastello et al. (2023)	Case study	Present a fictionalized case study that is an amalgamation of several patients with OCD, autism, and a gender diverse identity	Total N = 1 (ASD = 1; GD = 1)	Total Male = 0 (ASD = 0; GD = 0), Total Female = 1 (ASD = 1; GD = 1)	Total Gender-neutral = 1 (ASD = 1, GD = 1)	17 years	USA
Hillier et al. (2020)	Qualitative interview	Understand the first-hand experiences of autistic individuals who identify as LGBTQ +	Total N = 4 (ASD = 4; GD = 4)	NR	Total Male = 1 (ASD = 1; GD = 1), Total Nonbinary/Agender = 1 (ASD = 1; GD = 1), Total Nonbinary = 1 (ASD = 1; GD = 1), Total Queer = 1 (ASD = 1; GD = 1)	20 to 38 years	USA
Kourti and MacLeod (2019)	Qualitative interview	Illuminate autistic girls' exploration of their gender identities	Total N = 21 (ASD = 21; GD = NR)	Total Female = 21 (ASD = 21; GD = NR)	NR	21 to 52 years	International (UK, US, Canada, Norway, Germany, Republic of South Africa, Australia, New Zealand)
Longhurst et al. (2024)	Qualitative interview	Develop an understanding of positive body image in autistic adults in the UK, including how autistic adults experience their body image, how positive body image manifests among autistic adults, and characteristics of positive body image that are unique to autistic adults	Total N = 20 (ASD = 20; GD = NR)	NR	Total Male = 8 (ASD = 8; GD = NR), Total Female = 7 (ASD = 7; GD = NR), Total Nonbinary = 4 (ASD = 4; GD = 4), Total Agender = 1 (ASD = 1; GD = 1)	18 to 53 years	UK
Love et al. (2023)	Experience Sampling Methodology and Qualitative study	Utilize Experience Sampling Methodology to capture autistic people's thoughts, feelings, and behaviors surrounding opportunities to disclose one's diagnosis	Total N = 36 (ASD = 34; GD = NR)	NR	Total Male = 10 (ASD = NR; GD = NR), Total Female = 21 (ASD = NR; GD = NR), Total Nonbinary/Third Gender = 5 (ASD = NR; GD = 5)	21 to 71 years	International (Australia and UK)
Maroney et al. (2022)	Qualitative Interview	Explore the intersection of ASD and gender diverse identity	Total N = 13 (ASD = 13; GD = 13)	NR	Total Male = 2 (ASD = 2; GD = 2), Total Female = 2 (ASD = 2; GD = 2), Total Agender/Nonbinary = 5 (ASD = 5; GD = 5), Total Gender nonconforming = 1 (ASD = 1; GD = 1), Total Genderqueer = 1 (ASD = 1; GD = 1), Total Bigender = 1 (ASD = 1; GD = 1), Total Neuter = 1 (ASD = 1; GD = 1)	18 to 29 years	International (Canada and USA)
McAuliffe et al. (2022)	Qualitative interview	Examine how autistic LGBTQIA + individuals experience and understand their multiple minority identities and whether those identities impact their opportunities for participation in the LGBTQIA + community	Total N = 12 (ASD = 9; GD = 12)	NR	Total Male = 3 (ASD = 2; GD = NR), Total Female = 4 (ASD = 4; GD = NR), Total Non-Binary = 4 (ASD = 3; GD = 4), Total Agender = 1 (ASD = 0; GD = 1)	24 to 48 years	UK
Miller et al. (2019)	Qualitative interview	Explore how autistic LGBTQ college students make meaning of, manage, and express their LGBTQ and autistic identifies	Total N = 8 (ASD = 8, GD = 5)	NR	Total Male = 4 (ASD = 4; GD = 3), Total Female = 3 (ASD = 3, GD = 1), Total Nonbinary = 1 (ASD = 1; GD = 1)	Undergraduate and Graduates	USA
Parra (2022)	Qualitative interview	Present the experiences of the families of youth with gender diversity within the GAIA-Nueva Crianza Civil Association and families of Autistic youth within the Lazos Azules civil association	NR	NR	NR	NR	Argentina
Pham et al. (2021)	Case series	Identify trends in the presentation and management of disordered eating among transgender, autistic youth	Total N = 3 (ASD = 3; GD = 3)	Total Male = 2 (ASD = 2; GD = 2), Total Female = 1 (ASD = 1; GD = 1)	Total Male = 1 (ASD = 1; GD = 1), Total Female = 2 (ASD = 2; GD = 2)	14 to 17 years	USA
Shapira and Granek (2019)	Case studies and text analysis	Explore discourse around autism and GD from the perspective of researcher-clinicians and Autistic individuals	Total N = 13 (ASD = 11, GD = 9)	Total Male = 10 (ASD = 8; GD = 10), Total Female = 3 (ASD = 3; GD = 3)	Total Male = 3 (ASD = 3; GD = 3), Total Female = 6 (ASD = 6, GD = 6), Total Shifting Identification with Assigned Gender = 2 (ASD = 2; GD = 2), Total Stereotyped Feminine Behaviors = 2 (ASD = 2; GD = 2)	3 years and older	Canada and Israel
Strang et al., (2018a, 2018b, 2018c, 2018d)	Delphi	Establish clinical guidelines for assessment and care of adolescents with autism and GD	Total N = 22 (ASD = NR; GD = NR)	NR	NR	Adolescents	USA
Strang et al. (2019)	Case series	Provide vignettes on the autism/neurodiversity and gender diversity lived experience, emergence of identities, challenges faced by youth with the co-occurrence, and propose a community-based participatory research model for the autistic/neurodiverigent and gender diverse community	Total N = 9 (ASD = NR, GD = NR)	NR	NR	NR	International (US, UK, Europe, Canada)
Strang et al. (2020)	Community-based participatory research	Use a repeated-measures, community-based participatory research framework with neurodivergent, GD youth and families to identify care and support needs, develop interventions based on those needs, and receive feedback on interventions and study findings	Youth = 31 (ASD = 24; GD = 31); Parents = 46 (ASD = NR; GD = NR); Interpretation of finding consultants = 10 (ASD = NR; GD = NR), stakeholders, 10 experts (ASD = NR; GD = NR)	Youth Male = 16 (ASD = NR; GD = 16), Youth Female = 15 (ASD = NR; GD = 15); Parent Male = 16, Parent Female = 30	Youth Male = 11 (ASD = NR; GD = 11), Youth Female = 11 (ASD = NR; GD = 11), Youth Nonbinary = 4 (ASD = 4; GD = 4)	Youth: 12 to 19 years	USA
Valdez et al. (2022)	Case study	Illuminate the experience of an Autistic adolescent with gender diversity from a psychodynamic perspective	Total N = 1 (ASD = 1; GD = 1)	Total Male = 1 (ASD = 1, GD = 1)	Total Other = 1 (ASD = 1; GD = 1)	15 years	Chile
Zupanič et al. (2021)	Case Study	Present information on the assessment of gender diversity in an Autistic adolescent	Total N = 1 (ASD = 1; GD = 1)	Total Female = 1 (ASD = 1; GD = 1)	Total Male = 1 (ASD = 1; GD = 1)	16.5 years	Slovenia

NR, not reported; GD, gender diversity

#### Comparison with Øien et al. (2018) and Nordahl-Hansen et al. (2019)

The current review was statistically compared to the reviews by Øien et al. (2018) and Nordahl-Hansen et al. (2019) as there was overlapping search strategies and coding structures that allowed for comparisons.

#### Year

**Fig. 2:**
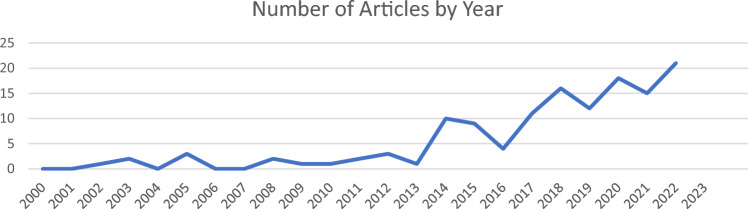
Number of articles published per year

As is apparent in Fig. 2, there was a sharp increase in studies on the intersection of gender identity and autism in 2014 and the number of studies has continued to increase about every other year since 2018.

#### Methodology

From August 2018 through January 2024, a majority of studies were quantitative (n = 69, 69.7%); 2 studies used mixed methods. There was not a significant difference between studies that were included in the original review and current studies in the use of qualitative versus quantitative methodologies based on a Fisher’s Exact Test (*p* = 0.270)*.* The most common specific methodologies were case–control (n = 43, 43.4%), survey studies (n = 20, 20.2%), and/or clinical chart reviews (n = 14, 14.1%). There were 7 longitudinal studies (7.1%) and 9 case studies (9.1%).

#### Age

**Fig. 3:**
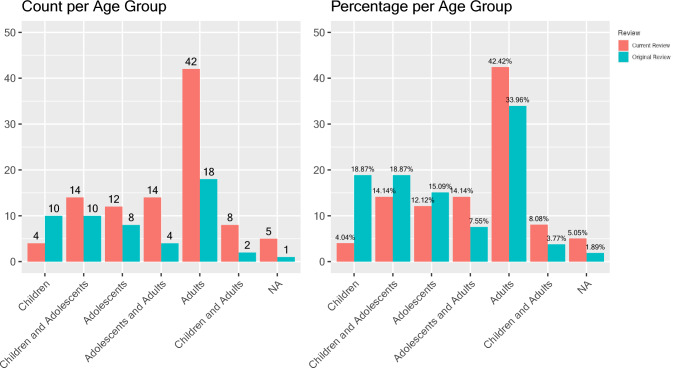
Number and percentage of studies representing each age group

There was not a statistically significant difference in the ages studied when comparing the original review and the current studies (*p* = 0.060). In both reviews (i.e., older and newer studies), the largest proportion of studies were conducted with adult participants (age 18 years and older) (n = 18, 34.0% for the original review; n = 42, 42.4% for the current review). By observation, there was a trend decrease in the number of studies that focused only on children (age 12 years and younger) and more studies that spanned age categories (e.g., included adolescents with adults or children, adolescents, and adults). See Table 4 for comparisons and Fig. 3 for distributions.

**Table 4:** Number of studies that focus on each age group presented in the original review (“Original”, 1981 through August 2018) and current review (“Current” after August 2018 through Dec 2022) as number of studies and percent of total (%)

Age group	1 Children	2 Adolescents	3 Adults	1&2	1&2&3	2&3	Not reported
Age in years	< 12	13–18	≥ 18	≤ 18		≥ 13	
1981–08/18 n = 53	10 (18.9%)	8 (15.1%)	18 (34.0%)	10 (18.9%)	2 (3.8%)	4 (7.5%)	1 (1.9%)
9/18–01/24 n = 99	4 (4.0%)	12 (12.1%)	42 (42.4%)	14 (14.1%)	8 (8.1%)	14 (14.1%)	5 (5.1%)

#### Participant Country of Origin

**Fig. 4:**
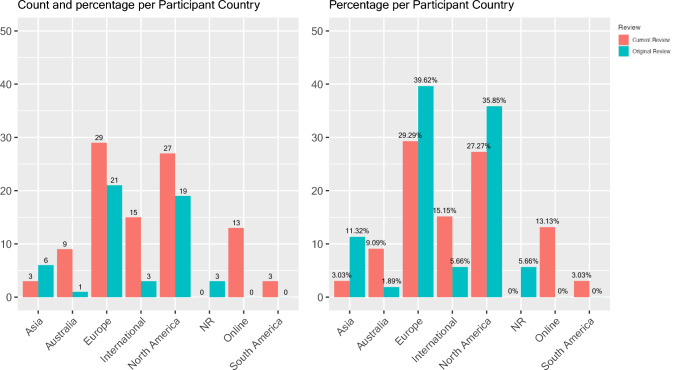
Number of studies representing participant country of origin

Among studies published after August 2018 and before or during January 2024, the research participants in studies were primarily from Europe (n = 29, 29.3%), North America (n = 27, 27.3%), or multiple countries (n = 15, 15.2%), as exhibited in Fig. 4. A Fisher’s Test indicated a significant difference between the Original Review (studies published prior to August 2018) and Current Review (August 2018 through January 2024) in the distribution of participant country (*p* = 0.0005), as displayed in Table 5. By observation, differences were likely driven by an increase in studies from Australia and South America, an increase in studies recruiting participants from multiple countries or online studies that did not specify country of participants, and a decrease in studies from Asia.

**Table 5:** Researchers’ and participants’ country of origin in each review

	Asia	Australia	Europe	North America	South America	International	Online
Participant country of origin							
1981–08/18 n = 50	6 (12.0%)	1 (2.0%)	21 (42.0%)	19 (38.0%)	0 (0%)	3 (6.0%)	NR
09/18–01/24 n = 99	3 (3.0%)	9 (9.1%)	29 (29.3%)	27 (27.3%)	3 (3.0%)	15 (15.2%)	13 (13.1%)
Researcher country of origin							
1981–08/18 n = 53	6 (11.3%)	4 (7.5%)	24 (45.3%)	19 (35.8%)	0 (0%)	0 (0%)	N/A
09/18–01/24 n = 99	1 (1.01%)	11 (11.1%)	35 (35.4%)	33 (33.3%)	3 (3.0%)	16 (16.1%)	N/A

3 studies did not report participant country of origin in the original review

#### Research Team Country of Origin

**Fig. 5:**
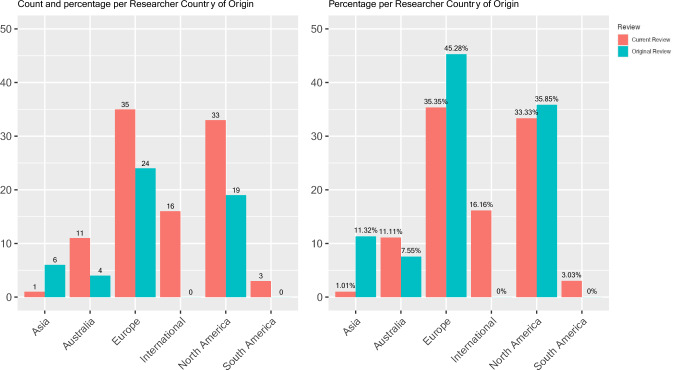
Number of studies representing researchers’ country of origin

There was also a significant difference between older and more recent studies regarding the country of origin of the researcher team (*χ*^2^(5) = 15.45, *p* = 0.009). See Table 4 for comparisons. For single country studies, most originated from Europe, North America, or Australia, as shown in Fig. 5. However, relatively more studies in recent years involved collaborations among researchers from more than one country; these studies are labeled “International” in Table 4 and included a European and North American country (n = 2) or a North American, European, and Asian country (n = 2).

### New Research Categories: Published Between August, 2018 Through January, 2024

Reflecting the expanded scope for the current review, we also abstracted the following characteristics of the recent studies: study research goals, study inclusion/exclusion criteria, autism diagnostic or trait measure used, gender characterization measure employed, assigned gender at birth and gender identity of participants, and inclusion of gender affirming care. None of these categories were coded in the original review.

#### Research Goals

Several studies had more than one aim and so were included in more than one category of research goals in the counts below. The most common aim of the studies was to identify common conditions faced by those at the intersection of autism and gender diversity (*n* = 36 studies, 36.4%). Other common foci of studies included: 1) experiential features of the autism and gender diversity intersection (*n* = 22, 22.2%) and 2) characterization of rates of autism diagnoses and/or the presence of autistic traits in gender diverse populations (*n* = 20, 20.2%). Relatively less common were studies on the prevalence of gender diverse identities among autistic samples (*n* = 12, 12.1%). Care and support needs were studied in 16 publications (16.2%) and perspectives of clinicians and parents were studied in 8 publications (8.1%). Other themes included sexuality and sexual experiences (*n* = 11, 11.1%) and gender diversity and autism from the perspective of the “extreme male brain theory” (*n* = 2, 2.0%). These themes were not always mutually exclusive.

#### Inclusion/Exclusion Criteria

The inclusion/exclusion criteria for studies ranged widely due to the different foci in the research. Inclusion/exclusion criteria were not reported in 17 studies (17.2%). For studies utilizing clinical data, participants were recruited from a range of settings, including gender diversity clinics, autism clinics, etc.; (*n* = 16, 16.2%). In 19 studies, participants were required to have an official or self-diagnosis of autism (19.2%). In 13 studies, participants were included if they had diagnoses of gender dysphoria (13.1%). Three studies (3.0%) attempted to include individuals with different communication preferences by offering communication or response accommodations (e.g., participants could complete an interview orally, in writing, or with an alternative communication device, depending on preference).

Regarding exclusion criteria, individuals with specific co-occurring conditions (e.g., psychosis) were excluded from 12 studies (12.1%) and individuals with intellectual disability were excluded from 9 studies (9.1%). Six studies (6.1%) restricted the range of gender diverse identities (e.g., excluded fluid gender identities or those who reported “other” gender). Common reasons for restricting gender categories were that there were too few participants in specific categories and/or studies had more power if they restricted analyses to a small number of groups.

#### Autism Trait Characterization or Diagnostic Confirmation

The most common method for ascertaining autism diagnosis was based on self-report (n = 36, 36.4%) or file review (n = 30, 30.3%). The self-report Autism Quotient (AQ; Baron-Cohen et al., 2001) was the primary measure used to measure autistic traits and/or used as diagnostic classification/confirmation (n = 25, 25.2%). The only other self- or parent-report measures of autism traits that were used with some regularity were the Social Communication Questionnaire (Rutter et al., 2003, n = 6, 6.1%) and the Social Responsiveness Scale (Constantino & Gruber, 2012, n = 11, 11.1%). Clinician administered/scored autism assessments included the Autism Diagnostic Observation Schedule, 2nd Edition (ADOS-2, Lord et al., 2012) (n = 10, 10.1%), the Autism Diagnostic Interview-Revised (ADI-R, Lord et al., 1994) (n = 8, 8.1%), or DSM/ICD checklists (n = 8, 8.1%).

#### Gender Identity Characterization

The method for characterizing gender identity was reported in 95 studies (96.0%). Eighteen different methods were used to characterize elements of gender diversity. The most common methods were to (1) ask participants their gender identity (n = 50, 50.5%) or (2) to conduct a medical or clinical file review of a gender dysphoria diagnosis (n = 25, 25.3%). The Gender Identity/Gender Dysphoria Questionnaire was used in 9 studies (9.1%). In three studies parents/caregivers were asked about their child’s gender (3.0%).

#### Gender Identity and Assigned Gender at Birth

A total of 27,182,721 participants were included in the current review. Of note, some studies were population studies (e.g., Chao et al., 2023), so the total number of participants is large. Additionally, some studies employed the standardization samples for measures without characterization of the standardization participants in terms of autism status or gender diversity (e.g., Kaltiala-Heino et al., 2019). Further, 21 of the studies did not report the number of individuals with autism diagnoses and 19 studies did not report the number of gender diverse individuals. Therefore, the proportion of autistic and gender diverse individuals compared to all individuals included across the studies is relatively low. As displayed in Table 6, 166,788 (0.61%) participants had diagnoses of autism, as reported in the studies, and 38,596 (0.14%) participants were described as being gender diverse. Neither assigned gender at birth nor gender identity were reported in 7 studies; 19 studies reported assigned gender at birth but not gender identity, and 27 studies reported gender identity but not assigned gender at birth. Only half of the studies (n = 46) reported both gender identity and assigned gender at birth.

**Table 6:** Sample sizes broken down by group, designated sex at birth, and gender

	Total N	Sex		Gender			
		Male	Female	Male	Female	Nonbinary	Other
Autistic	166,788	72,829	22,792	3345	2128	162	325
(n studies)	(n = 78)	(n = 40)		(n = 41)			
Gender diverse	38,596	10,282	11,551	4248	3924	2343	6288
(n studies)	(n = 80)	(n = 44)		(n = 54)			
Total	27,182,721	1,280,782	1,084,188	627,289	866,878	2343	6288
(n studies)	(n = 98)	(n = 65)		(n = 73)			

### Gender Affirming Care

There were 29 (29.3%) studies that reported on rates of individuals who were receiving and/or who had received gender affirming care. Of those, 16 studies (16.2%) reported specifically on rates of autistic, gender diverse individuals who received gender affirming care.

## Discussion

### Summary

This current review of publications on gender diversity and autism published between September 2018 and January 2024 provides an update to, and expansion of a previous review on gender diversity and autism (Nordahl-Hansen et al., 2019; Øien et al., 2018). Notably, there were about 1.5 times more studies published in the five and a half-year period after August 2018 through January 2024 as there were in the 37-year period from 1981 through August 2018, reflecting the growing research interest in this common intersection. The largest increase in publication occurred between 2016 and 2018, with other spikes occurring between 2019–2020 and 2021–2022. The increase in publications over the past 6 years parallels increased international recognition of the intersection of gender diversity and autism in clinical and community settings (Network AWN, 2023), as well as numerous WPATH international trainings on the clinical needs of autistic gender people (WPATH, 2018; WPATH, 2022).

The largest proportion of both recent and older publications present quantitative studies and studies focused on adults. Shifts in researcher and participant country of origin suggested a recent increase in international or multinational research, though with a decrease in studies from Asia. The broadening of locations of research participants and nationalities of research teams parallels recent increases in social-cultural conversations regarding gender diversity and gender identity, a trend which continues to expand globally (Shannon et al., 2019). The ability to safely reveal one’s gender diversity—and access the very terms/language associated with gender diversity—is not yet a universal; there are many countries and communities in which communicating gender diverse experiences would lead to punishment, and sometimes even death. The varying contexts regarding gender diversity across countries, as well as access to autism diagnostics, certainly drives some of disparities in reports from various global regions. However, there were numerous countries and communities not represented in the current research output that have more accepting attitudes and policies regarding gender diversity, and there is some evidence of trends toward greater acceptance in certain parts of the world (e.g., in parts of South America; Roberts, 2019). This may have facilitated the increase in the number of studies of the autism-gender diversity intersection within South American countries since the last review (i.e., 0–3).

Overall, research on the intersection of autism and gender diversity has continued to be primarily European- and North-American-focused both in participants and research teams. While online surveys allowed for broad international inclusivity, they were still constrained by language and other access issues (e.g., access to technology). Inconsistent reporting of country of residence of participants in online studies (n = 15) precluded investigation of demographic differences that may influence the experiences of gender diverse autistic people.

### Autistic Voices

A common goal of recent studies was to report on the prevalence of autism and/or autistic traits in gender diverse individuals and vice versa. Yet this type of research may not be a priority for autistic gender-diverse individuals (Strang et al., 2019). There has been a notable trend in recent years, particularly 2023, towards elucidating lived experiences and care needs of autistic gender diverse individuals. Collaborations with stakeholders will help set research priorities and agendas to ensure that study goals and design are reflective of the communities being described. Collaboration with autistic gender diverse co-researchers would also likely improve the quality of surveys and manuscripts to ensure that researchers and clinicians are accounting for important experiential and demographic factors and presenting them appropriately.

Nine studies explicitly excluded individuals with intellectual disability. Rates of inclusion of individuals with intellectual disability was impossible to discern in most studies and this subgroup is under-represented in this area of research (McPhate et al., 2021; Nunes-Moreno et al., 2022). Due to the lack of inclusion of individuals across a full range of cognitive and verbal abilities, key questions remain unanswered. For example, what is the proportion of autistic individuals with intellectual disability who are gender diverse? Do gender characterization approaches provide individuals with intellectual disability and/or significant communication differences the opportunity to appropriately report on their inner experience of gender? One way to potentially increase accessibility for individuals with intellectual disability or individuals with varied communication needs to participate and communicate gender would be to allow for choice in responding such as through oral, written, or alternative communication devices; this practice was only employed in two studies in the current review. Of note, a recent initiative using a community-based participatory process to characterize gender identity in autistic adults concluded that informant proxy report (i.e., report by a person who knows the autistic individual) of an individual’s gender identity may be ethically inappropriate (Nicolaidis, 2021). That is, reporting on inner experiences such as gender identity may be too personal and internal to understand through behaviors alone making proxy report highly suspect.

### Measurement Questions

There have been some methodological changes in the characterization of gender diversity and autism between the two reviews, but improvements are needed. Regarding gender, many recent studies have moved away from the single-item on the Child Behavior Checklist or Youth Self-Report, “wishes [wish] to be the opposite sex,” a measurement approach employed in many previous studies that has been criticized due to its binary nature (e.g., Manjra & Masic, 2022). The Gender Identity/Gender Dysphoria Questionnaire was used in several studies; this measure is reinforcing of the gender binary and contains stigmatizing language (e.g., “hermaphrodite” sic). Many studies also rely on a diagnosis of gender dysphoria, which represents only a subset of gender diverse individuals (e.g., individuals whose gender needs have not been addressed; de Vries et al., 2010). Future research could employ the US National Academy of Sciences’ recommended two-pass system for identifying gender diverse individuals; this approach asks first about gender identity and then assigned gender at birth in order to identify gender diverse participants (National Academies of Sciences, Engineering, and Medicine, 2022). An option for for further gender characteriziation is an open-ended textbox asking about a person’s gender; this provides participants with opportunities to offer their personal gender identity descriptors. However, such approaches might need to be combined with multi-dimensional continuous measurement of gender, as analytics based on multiple gender identity categories may result in overly small individual cell sizes for statistical analyses. A solution has been developed by Strang et al. (2023a, 2023b, 2023c, 2023d): “The Gender Self-Report,” calibrated in autistic and not autistic youth and adults, captures continuous non-binary and binary gender diversity factors, which can augment individuals’ self-descriptions of their gender and side step the small cell size challenge for analytics.

The characterization of autism and autistic traits also requires careful consideration. The self-report AQ remained the most commonly used measure in recent studies. But trait-based measures, such as the AQ, do not provide a clinical autism diagnosis, a process that includes an expert clinician evaluating an individual to characterize autism diagnosis or traits based on multi-modal information. Clinical assessments, which include, when possible, intervies and assessment of early developmental history, may help to distinguish autism traits from anxiety, depression, or minority-related stress. Emerging approaches for better characterizing autism in girls, women, and gender diverse individuals broadly may also be important, given late or missed diagnosis reported in a subset of individuals who are not cisgender male (Begeer et al., 2013; Lai, Lin, & Ameis, 2022; McQuaid et al., 2023a, 2023b, 2023c). Further, research will be needed on the use of existing state of the art autism assessments, as they were designed based primarily on cisgender male presentations of autism and may be less sensitive in other genders (Lai et al., 2022; Rea et al., 2023).

### Clinical Considerations

Only a minority of studies (n = 29) reported on gender affirming medical care received by samples. Yet, such treatments have been shown to improve mental health in gender diverse individuals who require them (e.g., Aldridge et al., 2021). Moving forward, researchers should consider the impact of various gender affirming care procedures on their findings, as well as potential challenges accessing such care, challenges which have been previously linked to autism status (i.e., unique care barriers experienced by autistic gender diverse individuals; Strang et al., 2018a, 2018b, 2018c, 2018d). There should also be more consideration for, and reporting of, other key aspects of gender affirmation, such as pathways and experienced barriers to social transition.

Age and developmental stage will be important to measure and model in future research designs as these factors have implications for clinical and medical recommendations. Extant research often groups together individuals at different developmental stages (e.g., adolescents and adults), which may obscure the impact of age and developmental stage on the presentation of—and needed supports for—gender diversity and autism. There has been increased attention on adult populations since the 2018 review, which is in keeping with an increasing recognition that autistic adults have been significantly under-represented in research, generally (Malik-Soni et al., 2021). In studies across developmental stages, it will be important to consider timing of autism diagnosis, including potential differences in timing of autism diagnosis for gender diverse individuals. There was a trend since the previous review toward fewer child focused studies. Longitudinal research on trajectories of autistic children as they become aware of their gender diverse identities as well as gender diverse individuals who come to know their autism is needed to understand how gender development, autistic development, and life experiences intersect and lead to patterns of resilience and/or risk.

### Limitations

This review reports on empirical studies of the intersection of gender diversity and autism published after August 2018 and not included in the previous 2018 review or update to the review. This review was not pre-registered, as Prospero does not allow for pre-registration of scoping reviews and this is an update to a previous review. We did not include unpublished studies as they were not peer-reviewed. Although no language restrictions were set, papers in various languages that do not provide English abstracts may not have been detected. The initial review published in 2018 did not code several important characteristics of studies and study samples, including gender of participants, gender and assigned gender at birth, method of characterizing autism or gender diversity, information on gender affirmation/transition, or exclusion criteria. Thus, those domains could not be compared between the older and the current reviews. We included these newly coded domains in the supplementary materials with the goal of supporting future scoping reviews with a broader scope. Future studies should also more equitably include stakeholders, with stakeholder collaborators representing a broad range of ethnoracial identities and lived experiences.

## Conclusion

This review described empirical studies of the intersection of gender diversity and autism since the 2018 review. Overall, this review indicated growth in the quantity and quality of studies in the field of gender identity and autism, although substantial improvement in methodologies is still needed. We encourage researchers to be sensitive in their use of terminology, measures, and methods. Research into the intersection of autism and gender diversity requires deep thoughtfulness and careful consideration of the real-world implications and impact of this research on the lives of individuals autistic gender-diverse people.

## Supplementary Information

Below is the link to the electronic supplementary material.

Supplementary file 1 (XLSX 137 KB)

Supplementary file 2 (XLSX 26 KB)

## Footnotes

1 Assigned gender at birth here is defined as biological and physiological characteristics often associated with being male, female, and/or having a variation of sex traits (also known as difference of sex development or intersex).

2 Identity first language is included in accordance with some autistic advocates’ stated preferences (Kenny et al., 2016).

## Glossary

Affirmed gender: Gender an individual wishes to be known by
Cisgender: A descriptor for people whose gender matches their assigned gender at birth
Gender diversity: When an individual’s gender identity or expression differs from cultural norms associated with assigned gender at birth
Gender fluid: A descriptor for people whose gender identity fluctuates
Gender dysphoria: DSM-5 diagnosis capturing psychological distress related to the discordance between one’s gender identity and one's sex designated at birth (APA, 2013; World Health Organization, 2018; 2021)
Gender identity: The inner experience of gender, such as whether one experiences themselves as nonbinary, male, female, agender, etc. (APA, 2015)
Gender incongruence: ICD-11 designation for difference between gender identity and assigned gender at birth
Gender nonbinary: Gender identity that is neither male or female
Medical gender affirmation: Medical approaches used to support an individual’s gender affirmation
Assigned gender at birth: The designation of sex that is made at birth based on apparent characteristics, typically related to the genitals
Transgender binary: A female or male gender identity that differs from an individual’s assigned gender at birth
Transgender female: An individual whose gender identity is female and who was designated male at birth
Transgender male: An individual whose gender identity is male and who was designated female at birth
